# Effectiveness of Generic and Disease-Specific Questionnaires in Assessing Quality of Life in Children With Type 1 Diabetes

**DOI:** 10.7759/cureus.83931

**Published:** 2025-05-11

**Authors:** Elmedina Mrkulic, Jasmina Mahmutovic, Sabina Terzic

**Affiliations:** 1 Pediatrics and Child Health, Pediatric Clinic, Clinical Center University of Sarajevo, Sarajevo, BIH; 2 Nursing, Faculty of Health Studies, University of Sarajevo, Sarajevo, BIH; 3 Pediatrics, Clinical Center University of Sarajevo, Sarajevo, BIH

**Keywords:** children and adolescents, diabetes specific measures, diabetes type 1, generic measures, health-related quality of life (hrqol)

## Abstract

Background: Health-related quality of life (HRQoL) evaluates the impact of health conditions on personal functioning. Type 1 diabetes mellitus (T1DM) in children and adolescents can significantly affect HRQoL due to the demands of daily disease management, psychological burden, and potential complications. The use of validated tools like Pediatric Quality of Life Inventory (PedsQL™) questionnaires is essential in assessing HRQoL. Combining generic and disease-specific scales offers a comprehensive evaluation.

Aim: The aim of this study is to assess the extent to which the PedsQL 4.0 and PedsQL 3.0 questionnaires explain the overall quality of life of children and adolescents with type 1 diabetes when used separately and in combination. Additionally, the study aims to identify which specific domains within these questionnaires are most significant in explaining the variability in overall quality of life scores.

Methods: A cross-sectional descriptive-analytical study was conducted from October 2021 to February 2022. The study assessed the quality of life of 50 children and adolescents (aged five to 18 years) diagnosed with type 1 diabetes mellitus, residing in Sarajevo Canton. Of these, 47 children/adolescents provided self-reports, while three children aged five to seven years were excluded from self-report analyses due to developmental limitations. However, parent proxy-reports were obtained for all 50 participants, including the three younger children. Accordingly, the final analysis included 47 child/adolescent self-reports and 50 parent proxy-reports. The study included children with a disease duration of >6 months and parental consent. The PedsQL™ 4.0 and PedsQL™ 3.0 Diabetes Module were used. Data analysis was performed using Statistical Package for Social Sciences (SPSS) version 26 (IBM Corp., Armonk, New York), including reliability testing, descriptive statistics, and multiple linear regression. Linear regression was selected to quantify the contribution of each questionnaire domain to overall HRQoL, offering an interpretable and robust method for assessing additive domain effects.

Results: The combined use of generic and diabetes-specific measures provided the most accurate assessment of HRQoL, capturing both general well-being and disease-specific challenges. Diabetes symptoms and physical functioning were key explainors.

Conclusion: The combined use of generic and diabetes-specific tools enables a more nuanced and comprehensive assessment of HRQoL in children and adolescents with T1DM. This study demonstrates that neither tool alone offers sufficient coverage, reinforcing the necessity of integrated assessment.

## Introduction

The assessment of quality of life (QoL) is most commonly based on the concept of health-related quality of life (HRQoL), as it encompasses various aspects of health status, including personal experiences, perceptions, functioning, and adaptation within different life domains [1]. Type 1 diabetes mellitus (T1DM) is one of the most common chronic diseases in childhood and adolescence. The presence of diabetes permanently alters the lives of affected individuals, increasing the risk of acute and chronic complications, which may lead to lower HRQoL [2]. While traditional health studies have focused on biomedical outcomes, HRQoL has become a key research concept in healthcare and medical practice over the past decades, significantly expanding its application in research and clinical practice [3]. It is recommended that psychosocial assessment be a routine part of the healthcare management of patients with diabetes, utilizing validated age-appropriate tools. The implementation of these tools in clinical practice would enable monitoring and evaluation of the overall psychosocial well-being and QoL of children and adolescents with diabetes [4]. Haraldstad et al. emphasize that knowledge about quality of life is crucial for understanding the impact of disease and treatment and for making medical decisions. They also point out that, although quality of life is an important aspect of medical research, many studies face methodological and conceptual challenges, indicating the need for improvements in this field. A crucial step in QoL assessment is the appropriate selection of questionnaires. Frequently used instruments for measuring HRQoL in children and adolescents with T1DM are Pediatric Quality of Life Inventory (PedsQL) questionnaires [3], which were also used in this study.

Generic QoL measures can be used independently or in combination with disease-specific instruments, depending on the research objectives. While generic measures assess physical, emotional, and social well-being, disease-specific QoL instruments provide unique insights that generic measures do not cover. The psychometric properties of each measure, along with their relationships to relevant outcomes, support the use of both the PedsQL generic and diabetes module in QoL assessment. The combined use of both measurement scales is considered an ideal approach for assessing HRQoL in patients with T1DM, incorporating the benefits of both generic and disease-specific instruments [5,6]. The use of generic and diabetes-specific HRQoL assessments is often advocated for their utility in research. Findings from studies examining the HRQoL of children and adolescents with diabetes can significantly contribute to professional practice development and improve the quality of healthcare services for children and adolescents with T1DM, particularly in low- and middle-income countries [7].

A review of published studies on the quality of life of children and adolescents using PedsQL measurement scales (the generic scale and the diabetes module) revealed different approaches in applying these scales to assess the QoL of children and adolescents with T1DM. In several studies, both measurement scales were used [5,8-10] without summing the total score of both scales, while in other studies, only the generic scale [2,7,11-13] or only the diabetes module [14-20] was used, depending on the study objectives. Given that diabetes significantly affects the daily activities of children and adolescents, and that the generic and diabetes module scales encompass different aspects of quality of life, this study integrated both measurement scales by summing the total score of both scales. 

The aim of this study is to assess the extent to which the PedsQL 4.0 and PedsQL 3.0 questionnaires explain the overall quality of life of children and adolescents with type 1 diabetes when used separately and in combination. Additionally, the study aims to identify which specific domains within these questionnaires are most significant in explaining the variability in overall quality of life scores.

## Materials and methods

The study included children and adolescents (aged five to 18 years) with a confirmed diagnosis of type 1 diabetes mellitus (T1DM), who are permanent residents of Sarajevo Canton and members of the Association of Diabetic Children and Youth of Sarajevo Canton. After obtaining approval from the Diabetes Association, the study was offered to 69 available parents of children and adolescents with T1DM. Out of a total of 69 contacted parents, 51 initially agreed to participate in the study with their child/adolescent, while 18 parents declined participation. One participant was subsequently excluded from the study because the duration of the disease was less than six months. Therefore, the final number of participants included in the study was 50, representing a participation rate of 72.46%.

Inclusion criteria were: disease duration longer than six months, age between five and 18 years at the time of participation, and voluntary written informed consent provided by a parent or legal guardian. One parent (primary caregiver) of each child/adolescent was also included in the study to provide a proxy assessment of the child's quality of life.

Exclusion criteria included a disease duration of less than six months, lack of parental consent, and inadequate reliability of child self-reports based on internal consistency analysis. Specifically, although 50 children and adolescents were enrolled, only 47 self-reports were included in the final analysis. Three children aged five to seven years were excluded from the self-report component due to developmental limitations and low internal consistency scores of their responses. The overall quality of life score in this age subgroup yielded a Cronbach's alpha of 0.424. The generic core scale showed an alpha of 0.623, while the diabetes-specific module yielded a coefficient of 0.291. As these values fall below commonly accepted thresholds for reliability (α < 0.70), the self-reports of this age group were excluded from the analysis.

Nevertheless, proxy reports from parents were obtained and analyzed for all 50 participants. Parental reports are especially valuable for younger children, where cognitive and developmental factors may limit the accuracy of self-assessment.

Pediatric Quality of Life Inventory 4.0 (PedsQL™ 4.0) is a generic instrument for measuring the quality of life in children and adolescents aged two to 18 years. PedsQL™ 4.0 consists of 23 items divided into four dimensions: physical functioning (eight items), emotional functioning (five items), social functioning (five items), and school/kindergarten functioning (five items). The questionnaire is designed for children with acute and chronic health conditions, as well as for healthy children. The child and adolescent questionnaires are based on a Likert scale response format (0=never, 1=almost never, 2=sometimes, 3=often, 4=almost always), where the scores are linearly transformed to a scale of 0-100, with higher scores indicating better quality of life [21].

PedsQL™ 3.0 Diabetes Module is a disease-specific instrument for measuring quality of life in children and adolescents with diabetes. It includes 28 items divided into five subscales: diabetes symptoms (11 items), treatment barriers (four items), treatment adherence (seven items), worry (three items), and communication (three items). The diabetes questionnaires use the same Likert scale and scoring method as PedsQL™ 4.0. Both measurement instruments include self-reports from children and adolescents as well as proxy reports from parents [5,22].

Permission and a license for using the questionnaires in this study were obtained from Mapi Research Trust. The questionnaires were available in the official languages of Bosnia and Herzegovina.

The study was conducted within the Association of Children and Youth with Diabetes in Sarajevo Canton from October 23, 2021, to February 28, 2022. After the Association granted approval, data on members were accessed, and interviews with participants were scheduled. The research was conducted in carefully selected locations convenient for participants, including public spaces and private homes. Each participant had an individual session. After obtaining written consent, participants proceeded with completing the questionnaires. Children aged five to seven years completed the questionnaires with the help of the researcher, while children aged eight years and older completed them independently, with minimal assistance from the researcher. Parents/caregivers completed their questionnaires independently, with researcher assistance if they had any uncertainties. Completing the questionnaires took an average of about 10 minutes.

During the research, the Ethical Code of Research with children and on children in Bosnia and Herzegovina was respected. This code, in accordance with the UN Convention on the Rights of the Child, aims to protect children from any form of abuse. It regulates the status of children and their families as participants in various studies--humanitarian, social, educational, medical, and others--that may impact their personal integrity [23].

Statistical analysis was performed using IBM SPSS Statistics version 26.00 (IBM Corporation, Armonk, New York). The reliability of the questionnaires was assessed using Cronbach's alpha test. Descriptive analysis results were presented using mean values and standard deviations with a 95% confidence interval. Multiple linear regression was applied to examine and model the relationship between one dependent variable (Y) and one or more independent variables (X). The IBM SPSS Automatic Linear Modeling module was used to identify the factors that significantly influence the quality of life scores of children and adolescents with type 1 diabetes. Pearson's correlation coefficient was used to examine the interrelationship between subscales, where higher values indicate a stronger association between variables. Statistical significance was set at p < 0.05.

## Results

The quality of life analysis included 47 (94%) self-reports from children and adolescents and 50 (100%) caregiver proxy reports. Consequently, further analyses of quality of life were conducted using self-reports from children and adolescents aged 8-18 years n=47, and parent proxy reports for children/adolescents aged five to 18 years n=50 (Table 1).

**Table 1:** Characteristics of study participants and caregivers.

Characteristics	n	%
Age group: children and adolescents		
5–7 years	3	6
8–12 years	22	44
13–18 years	25	50
Total	50	100
Primary caregiver		
Mother	49	98
Father	1	2
Total	50	100

Table 2 presents the assessment of quality of life in children with type 1 diabetes based on both self-reports and parent proxy-reports, including total and domain-specific scores, as well as the internal consistency of the questionnaire (Cronbach's alpha).

**Table 2:** Assessment of quality of life in children with type 1 diabetes and internal consistency of the questionnaire. "α" refers to Cronbach's alpha coefficient, which indicates the internal consistency (reliability) of each subscale. The child self-report includes data from 47 participants; self-reports from three children aged five to seven years were excluded from the analysis due to developmental limitations affecting questionnaire comprehension. Nevertheless, proxy reports from their parents were included in the overall assessment (N=50). Internal consistency checks conducted across age groups revealed that the reliability of self-reported data in the five to seven-year age group was inadequate. Specifically, the overall quality of life score in this subgroup yielded a Cronbach’s alpha of 0.424, the generic core scale a coefficient of 0.623, and the diabetes-specific module a coefficient of 0.291. Due to the insufficient reliability of these measures, the self-reports from this age group were excluded from the final analysis.

Child self-report (N=47)	Number of items	Mean	SD	95% CI	α	
Quality of life with type 1 diabetes mellitus total score	51	79.81	9.54	76.7-82.16	0.879	
Generic total score	23	82.77	9.16	80.08-85.46	0.812	
Physical health	8	84.84	11.89	81.35-88.33	0.746	
Psychosocial health	15	81.67	10.13	78.69-84.64	0.758	
Emotional functioning	5	72.87	16.57	68.01-77.74	0.651	
Social functioning	5	95.21	8.14	92.82-97.6	0.641	
School functioning	5	76.92	14.70	72.61-81.24	0.634	
Diabetes module total score	28	77.68	11.87	74.20-81.17	0.817	
Diabetes symptoms	11	69.73	14.55	65.46-74.0	0.811	
Treatment barriers	4	75.13	18.13	69.81-80.46	0.604	
Treatment adherence	7	87.88	13.22	84.00-91.76	0.618	
Worry	3	73.94	19.71	68.15-79.72	0.612	
Communication	3	81.74	22.43	75.15-88.32	0.724	
Parent proxy-report, N=50						
Quality of life with type 1 diabetes mellitus total score	51	76.27	13.69	72.38-80.16	0.934	
Generic total score	23	78.83	15.23	74.50-83.15	0.926	
Physical health	8	83.06	18.34	77.85-88.28	0.883	
Psychosocial health	15	76.57	15.28	72.22-80.91	0.885	
Emotional functioning	5	67.20	19.51	61.65-72.75	0.786	
Social functioning	5	87.80	16.76	83.04-92.56	0.858	
School functioning	5	74.70	19.23	69.23-80.17	0.791	
Diabetes module total score	28	74.76	14.62	70.60-78.91	0.868	
Diabetes symptoms	11	68.05	16.33	63.40-72.69	0.869	
Treatment barriers	4	79.38	19.53	73.82-84.93	0.721	
Treatment adherence	7	83.87	15.26	79.53-88.21	0.681	
Worry	3	70.33	23.88	63.55-77.12	0.777	
Communication	3	72.17	26.55	64.62-79.71	0.859	

Table 3 presents the intercorrelations between the diabetes module subscales and the total quality of life (TQL), generic scale (GS), and individual domains of quality of life (physical, psychosocial, emotional, social, and school functioning).

**Table 3:** Intercorrelation of diabetes module subscales with the total quality of life score and generic scale with subscales. The statistical test used to analyze the p-values is Pearson's correlation coefficient for assessing the strength and direction of linear relationships. r represents the correlation coefficient, which indicates the strength and direction of the relationship between the variables. p represents the p-value, which tests the statistical significance of the correlation; values less than 0.05 indicate statistically significant relationships. TQL: total quality of life, GS: generic scale, PF: physical functioning, PSZ: psychosocial health, EF: emotional functioning, SF: social functioning, SchF: school functioning. *p < 0.05.

Children's and adolescent's reports		TQL	GS	PF	PSZ	EF	SF	SchF
Diabetes symptoms	r	0.821	0.602	0.436	0.560	0.429	0.205	0.555
	p	<0.001*	<0.001*	0.002*	<0.001*	0.002*	0.153	<0.001*
Treatment barriers	r	0.525	0.306	-0.003	0.423	0.244	0.290	0.431
	p	<0.001*	0.031*	0.986	0.002*	0.087	0.041*	0.002*
Treatment adherence	r	0.693	0.452	0.423	0.421	0.268	0.149	0.481
	p	<0.001*	0.001*	0.002*	0.002*	0.059	0.300	<0.001*
Worry	r	0.479	0.289	0.099	0.337	0.321	0.193	0.224
	p	<0.001*	0.042*	0.496	0.017*	0.023*	0.180	0.118
Communication	r	0.485	0.205	0.103	0.218	0.109	0.143	0.245
	p	<0.001*	0.154	0.476	0.128	0.453	0.322	0.086
Parent's proxy reports								
Diabetes symptoms	r	0.855	0.739	0.609	0.740	0.641	0.556	0.628
	p	<0.001*	<0.001*	<0.001*	<0.001*	<0.001*	0.001*	<0.001*
Treatment barriers	r	0.714	0.546	0.391	0.584	0.629	0.369	0.431
	p	<0.001*	<0.001*	0.005*	<0.001*	<0.001*	0.008*	0.002*
Treatment adherence	r	0.774	0.669	0.565	0.660	0.523	0.645	0.480
	p	<0.001*	<0.001*	<0.001*	<0.001*	<0.001*	<0.001*	<0.001*
Worry	r	0.677	0.547	0.467	0.536	0.543	0.420	0.362
	p	<0.001*	<0.001*	0.001*	<0.001*	<0.001*	0.002*	0.010*
Communication	r	0.336	0.189	0.181	0.173	0.168	0.192	0.073
	p	0.017*	0.189	0.208	0.231	0.243	0.181	0.613

For the children's and adolescents' report, diabetes symptoms show the strongest correlations with both TQL and GS. The results indicate that diabetes symptoms and treatment adherence have the strongest associations with overall quality of life.

Table 4 presents regression models analyzing the contribution of various subscales from the questionnaires to explaining quality of life in children with type 1 diabetes.

**Table 4:** Regression models for assessing the effectiveness of generic and diabetes-specific questionnaires in explaining quality of life in children with T1DM, both independently and in combination. The statistical test used to analyze the p-values is multiple linear regression. This method was employed to assess the relationship between the explainors (various subscales) and the outcome variables (quality of life, generic score, and diabetes module score). The p-values indicate the statistical significance of each regression model, with values less than 0.05 considered statistically significant. The regression models assess the impact of multiple explainors on the overall quality of life and subscale scores for both children's self-reports and parent proxy-reports. Model 1: Regression model predicting overall quality of life based on the generic and diabetes module subscales. Predictors: (Constant), Diabetes symptoms, Treatment barriers, Treatment adherence, Worry, Communication, Physical functioning, Emotional functioning, Social functioning, School functioning. Model 2: Regression model predicting the generic score based on the subscales of the generic questionnaire. Predictors: (Constant), Physical functioning, Emotional functioning, Social functioning, School functioning. Model 3: Regression model predicting the diabetes module score based on the subscales of the diabetes module. Predictors: (Constant), Diabetes symptoms, Treatment barriers, Treatment adherence, Worry, Communication. Model 4: Regression model predicting overall quality of life based on the subscales of the generic questionnaire. Predictors: (Constant), Physical functioning, Emotional functioning, Social functioning, School functioning. Model 5: Regression model predicting overall quality of life based on the subscales of the diabetes module. Predictors: (Constant), Diabetes symptoms, Treatment barriers, Treatment adherence, Worry, Communication. Model 6: Regression model predicting the generic score based on the subscales of the diabetes module. Predictors: (Constant), Diabetes symptoms, Treatment barriers, Treatment adherence, Treatment concerns, Communication.

Children's self-report: Regression models	R	R²	Adjusted R²	SD	F	df1	df2	p
Model 1	1.000	1.000	1.000	0.121	30767.93	9	40	<0.0001
Model 2	0.999	0.999	0.999	0.000	2.027 × 10^16^	4	45	<0.0001
Model 3	0.999	0.996	0.996	1.100	1093.468	5	44	<0.0001
Model 4	0.871	0.759	0.737	4.697	35.385	4	45	<0.0001
Model 5	0.939	0.882	0.869	3.319	65.925	5	44	<0.0001
Model 6	0.645	0.416	0.349	7.312	6.263	5	44	<0.0001
Parent proxy report: Regression models								
Model 1	1.000	1.000	1.000	0.106	90455.41	9	40	<0.0001
Model 2	1.000	1.000	1.000	0.00287	34434 × 10^3^	4	45	<0.0001
Model 3	1.000	1.000	1.000	0.00287	34434 × 10^3^	4	45	<0.0001
Model 4	0.945	0.893	0.884	4.663	94.257	4	45	<0.0001
Model 5	0.956	0.914	0.904	4.247	92.943	5	44	<0.0001
Model 6	0.813	0.661	0.622	9.358	17.146	5	44	<0.0001

As shown in Table 5, the table presents the results of regression models identifying factors that significantly influence the quality of life scores of children and adolescents with type 1 diabetes. The analysis highlights the importance of physical, school, and emotional functioning in children's self-reports and parent proxy-reports. Additionally, key diabetes-related factors, such as communication, treatment barriers, worry, and treatment adherence, make a significant relative contribution in explaining variations in quality of life outcomes.

**Table 5:** Factors influencing the generic and diabetes-specific quality of life scores in children and adolescents with type 1 diabetes. β represents the standardized regression coefficient, which indicates the strength and direction of the relationship between each factor and the dependent variable. t is the t-statistic, which measures the ratio of the difference between the observed and expected values to the standard error of the coefficient. The statistical test used to analyze the p-values is multiple linear regression, which evaluates the relationship between the independent variables (subscales) and the dependent variables (generic score and diabetes module score) for both children's and adolescents' self-reports and parent proxy-reports with values less than 0.05 considered statistically significant.

Dependent variable	Factor	β	S.E.	t	p	Relative contribution
Generic score-children's and adolescents' report	Constant (intercept)	-1.252	1.211	-1.034	0.307	-
	Physical functioning	0.386	0.010	36.787	<0.001	0.406
	School functioning	0.216	0.007	29.963	<0.001	0.269
	Emotional functioning	0.210	0.007	29.207	<0.001	0.256
	Social functioning	0.202	0.013	15.145	<0.001	0.069
Generic score-parents' proxy report	Constant (intercept)	-0.632	0.198	-3.189	0.003	-
	Physical functioning	0.350	0.003	118.12	<0.001	0.386
	Emotional functioning	0.216	0.002	103.10	<0.001	0.294
	School functioning	0.221	0.003	85.002	<0.001	0.200
	Social functioning	0.220	0.003	65.539	<0.001	0.119
Diabetes module score-children's and adolescents' report	Constant (intercept)	-1.344	1.662	-0.809	0.423	-
	Communication	0.203	0.010	19.550	<0.001	0.298
	Treatment barriers	0.201	0.011	17.698	<0.001	0.244
	Worry	0.184	0.011	16.826	<0.001	0.221
	Diabetes symptoms	0.213	0.015	13.909	<0.001	0.151
	Treatment adherence	0.212	0.020	10.487	<0.001	0.086
Diabetes module score-parents' proxy report	Constant (intercept)	-2.162	0.678	-3.190	0.003	-
	Communication	0.197	0.005	41.52	<0.001	0.406
	Worry	0.217	0.007	32.962	<0.001	0.256
	Treatment barriers	0.201	0.008	23.680	<0.001	0.132
	Diabetes symptoms	0.209	0.010	21.285	<0.001	0.107
	Treatment adherence	0.203	0.010	20.520	<0.001	0.099

Table 6 presents the regression coefficients, standard errors, t-values, p-values, and predictor importance for factors influencing the overall score of quality of life in children and adolescents with type 1 diabetes.

**Table 6:** Importance of factors in explaining the overall quality of life of children and adolescents with type 1 diabetes mellitus. β represents the standardized regression coefficient, which quantifies the strength and direction of the relationship between each explainor factor and the overall quality of life score. t is the t-statistic, which assesses the ratio of the difference between the observed and expected values to the standard error of the coefficient. The statistical test used to analyze the p-values is multiple linear regression, with values less than 0.05 considered statistically significant. This test examines the impact of various factors (such as diabetes symptoms, physical functioning, emotional functioning, etc.) on the overall quality of life scores, based on both children's and adolescents' self-reports and parent proxy-reports.

Dependent variable	Factor	β	S.E.	t	p	Relative contribution
Overall score quality of life children's and adolescents' report	Constant (intercept)	-1.178	0.733	-1.606	0.116	-
	Diabetes symptoms	0.221	0.006	38.481	<0.001	0.314
	Physical functioning	0.173	0.006	26.904	<0.001	0.153
	Emotional functioning	0.096	0.004	22.553	<0.001	0.108
	Treatment barriers	0.080	0.004	20.436	<0.001	0.088
	School functioning	0.098	0.005	20.410	<0.001	0.088
	Treatment adherence	0.129	0.007	19.429	<0.001	0.080
	Worry	0.064	0.003	18.303	<0.001	0.071
	Communication	0.058	0.003	17.600	<0.001	0.066
	Social functioning	0.095	0.008	12.383	<0.001	0.032
Overall score quality of life parent's proxy report	Constant (intercept)	-1.283	0.316	-4.063	<0.001	-
	Diabetes symptoms	0.223	0.005	43.974	<0.001	0.264
	Physical functioning	0.163	0.004	37.015	<0.001	0.186
	Communication	0.059	0.002	28.556	<0.001	0.111
	Emotional functioning	0.098	0.004	26.314	<0.001	0.094
	School functioning	0.094	0.004	24.624	<0.001	0.082
	Treatment adherence	0.118	0.005	23.402	<0.001	0.074
	Worry	0.069	0.003	22.742	<0.001	0.070
	Social functioning	0.109	0.005	21.002	<0.001	0.060
	Treatment barriers	0.082	0.004	20.874	<0.001	0.059

## Discussion

Research on quality of life (QOL) is complex due to multiple factors influencing its definition, measurement, and interpretation. Although QOL is increasingly used as an indicator in assessing health and social policies, its measurement is not always adapted for identifying vulnerable groups or evaluating the impact of interventions at an individual level, posing challenges in the application of research findings.

While the literature suggests that differences in quality of life perception between children and parents underscore the importance of using multiple sources of information when assessing the impact of type 1 diabetes on the daily lives of children and adolescents [5], it is possible that discrepancies between child and parent reports lead to inaccurate measurements [24].

The results of this study also reveal differences in the assessment of children's and adolescents' quality of life between parental reports and self-reports by children/adolescents. Considering that mothers, who are predominantly the primary caregivers, as confirmed by this study, are often emotionally affected by their child's illness [25,26], it is worth exploring the possibility that parents' subjective reports on their children's/adolescents' quality of life may be influenced by the mother's emotional state and her own quality of life. Future research should investigate this aspect. Although parents are often considered to provide a more objective assessment of their child’s quality of life [5], QOL remains a subjective evaluation of an individual’s development in various life domains [1]. Therefore, we believe that priority should be given to self-reports by children and adolescents, as their subjective assessment provides a more direct reflection of their own quality of life.

The exclusion of self-reports from children aged five to seven years was based on low internal consistency scores, reflecting potential developmental and cognitive limitations in this age group. This highlights the challenges of relying on self-report instruments in early childhood, where limited verbal skills and abstract reasoning may affect the reliability of responses.

A critical review by Wallander et al. (2016) highlights inconsistencies in the conceptualization and measurement of children's quality of life, particularly in differentiating between QOL indicators and their determinants, emphasizing the issue of discrepancies between subjective and objective assessments of QOL [24].

Measurement of quality of life is based on two main approaches: generic and specific instruments [27]. Generic instruments provide a comprehensive assessment of health-related quality of life (HRQoL), while specific instruments focus on particular health conditions, specific patient groups, or areas of healthcare [28]. Generic instruments assess constructs or feelings relevant to all individuals but do not account for daily activities specific to diabetes management. Personal experiences with diabetes may be influenced by various social and cultural factors, as well as individual factors such as education, access to information, healthcare support, and medical treatment [29]. They can be useful for broader population health research, whereas specific instruments better measure the particular aspects of life for children with certain health conditions [27]. In addition to the generic dimensions of physical, emotional, and social well-being, the diabetes module offers unique perspectives not covered by the generic scale [5]. These approaches are not mutually exclusive but are applied depending on the research context [28].

Our hypothesis, grounded in established theoretical frameworks, underscores the necessity of using both generic and disease-specific instruments to evaluate the quality of life (QoL) in children and adolescents with type 1 diabetes (T1DM). By integrating these two approaches, a comprehensive total score was created, reflecting the overall QoL across all relevant domains.

The findings of this study emphasize that diabetes-specific factors such as symptoms, treatment adherence, and treatment barriers are more strongly correlated with overall QoL (TQL) than with the generic scale (GS), as reported by both children/adolescents and their parents. This disparity is particularly evident in the case of diabetes symptoms, which demonstrated a stronger association with the total quality of life (TQL) score than with the generic scale (GS), highlighting their greater relevance in capturing the specific burden of the disease. Also, communication was statistically significantly related only to TQL in both reports, and not to GS, contrary to the findings of Girma et al., who suggested that better communication enhances general QoL scores [10].

Regression analyses were employed to evaluate the explanatory strength of different subscales for both generic and disease-specific instruments, and to identify the most influential components in assessing QoL. The integrated model, combining subscales from both instruments, outperformed all others. It achieved the highest values for correlation coefficients (R), explained variance (R² and adjusted R²), a very low standard error, and a high F-statistic, indicating superior explanatory accuracy. These results support a multidimensional assessment strategy that simultaneously accounts for general daily functioning (physical, emotional, social, and school) and disease-specific challenges, offering a more nuanced and realistic understanding of how diabetes affects children's lives.

While models using only generic measures provided statistically robust results, they lacked specificity in capturing individual disease-related difficulties. Similarly, models based solely on diabetes-specific measures were effective in elucidating disease-related experiences (e.g., symptom burden, treatment barriers, and emotional concerns), but failed to account for broader aspects of well-being. Notably, attempts to explain generic QoL scores using only diabetes-specific variables yielded significantly lower coefficients of determination, underscoring that aspects like emotional and social functioning are not strongly tied to direct disease indicators.

The integration of both instruments not only enhanced statistical accuracy but also deepened the understanding of how diabetes influences various life domains. Although children and adolescents emphasized certain domains more than their parents, the overall pattern of explainors remained consistent across informants. Diabetes symptoms and physical functioning emerged as the strongest explainors, yet other subscales also contributed meaningfully to QoL outcomes. These findings confirm previous research indicating that generic instruments alone lack sufficient sensitivity in chronic conditions [29], while disease-specific tools may miss broader psychosocial dimensions [5].

Given the all-encompassing nature of T1DM and the strict demands of its management regimen [30], it is essential to evaluate QoL through both standard and disease-specific lenses. This dual-perspective approach allows for a more accurate representation of the burden that diabetes imposes on daily life, adaptation, and treatment management.

The limitations of this study primarily relate to the sample size and geographic coverage. The research was conducted with a relatively small sample of 50 patients from the Sarajevo Canton, which may limit the generalizability of the findings to a broader population of children and adolescents with type 1 diabetes. The specific social, economic, and healthcare factors characteristic of this region could influence the results, and similar studies conducted in other regions or countries may reveal different patterns in quality of life. Additionally, differences in research methodology and the questionnaires used in other studies may make direct comparisons challenging. While validated instruments for measuring quality of life were used in this study, cultural differences in the perception of health and well-being might lead to variations in how children and their parents report their experiences. These limitations should be considered when interpreting the results, as they could affect the external validity of the findings. Furthermore, the small sample size may affect the reliability of the data, potentially leading to less stable estimates of the relationships between the predictors and the quality of life outcomes. A larger, more diverse sample would be beneficial in future research to enhance the generalizability and reliability of the findings.

## Conclusions

The integration of generic and disease-specific measures offers the most valid and comprehensive method for assessing health-related quality of life in pediatric diabetes. The results support the development of a unified measurement tool that aggregates all domains from both instruments into a single total score reflecting overall QoL in children and adolescents with T1DM. This study shows that combining generic and diabetes-specific questionnaires provides a more complete and accurate assessment of HRQoL in children and adolescents with type 1 diabetes than using either instrument alone. Future research should adopt this integrated approach to further clarify the multidimensional impact of diabetes and inform more tailored healthcare and psychosocial interventions. Although limited by sample size and regional scope, the study provides a strong foundation for future research and the design of targeted interventions that improve key HRQoL domains in pediatric diabetes care.
